# Mutational Biases Influence Parallel Adaptation

**DOI:** 10.1093/molbev/msx180

**Published:** 2017-06-22

**Authors:** Arlin Stoltzfus, David M. McCandlish

**Affiliations:** 1 Genome-scale Measurements Group, Material Measurement Laboratory, NIST, and Institute for Bioscience and Biotechnology Research, Rockville, MD 20850, USA; 2 Simons Center for Quantitative Biology, Cold Spring Harbor Laboratory, Cold Spring Harbor, NY 11724, USA

**Keywords:** mutation-biased adaptation, transition-transversion bias, parallelism, experimental evolution

## Abstract

While mutational biases strongly influence neutral molecular evolution, the role of mutational biases in shaping the course of adaptation is less clear. Here we consider the frequency of transitions relative to transversions among adaptive substitutions. Because mutation rates for transitions are higher than those for transversions, if mutational biases influence the dynamics of adaptation, then transitions should be overrepresented among documented adaptive substitutions. To test this hypothesis, we assembled two sets of data on putatively adaptive amino acid replacements that have occurred in parallel during evolution, either in nature or in the laboratory. We find that the frequency of transitions in these data sets is much higher than would be predicted under a null model where mutation has no effect. Our results are qualitatively similar even if we restrict ourself to changes that have occurred, not merely twice, but three or more times. These results suggest that the course of adaptation is biased by mutation.

## Introduction

Cases of parallel and convergent evolution frequently are invoked as displays of the power of selection. However, the influence of mutational biases on parallel evolution has not been considered until recently (Chevin et al. 2010; Lenormand et al. 2016; Storz 2016; Bailey et al. 2017). Classical arguments (reviewed by Yampolsky and Stoltzfus 2001) suggest that any influence of mutational bias should be overwhelmed in the presence of natural selection, on the grounds that mutation rates are very small and therefore have only a minor effect on changes in allele frequency (e.g., Fisher 1930, Chapter 1). Yet, in many common models of molecular adaptation the rate at which an adaptive allele becomes fixed in a population is directly proportional to its mutation rate (see McCandlish and Stoltzfus 2014, for a review). This effect arises due to a “first come, first served” dynamic that occurs when adaptive mutations appear in the population sufficiently infrequently. In particular, when adaptive mutations are rare, the first one to reach a substantial frequency is likely to become fixed, whereas when adaptive mutations are common, the fittest mutation is most likely to fix regardless of mutational biases (Yampolsky and Stoltzfus 2001). Because the impact of mutational biases depends sensitively on prevailing population-genetic conditions, determining the importance of mutational biases for parallel adaptation is necessarily an empirical issue.

The most direct evidence for a pervasive role of mutational biases in adaptation comes from experimental studies that manipulate the mutational spectrum and observe the effects on the outcome of adaptation. For instance, Cunningham et al. (1997) adapted bacteriophage T7 in the presence of the mutagen nitrosoguanidine. Deletions evolved 9 times and nonsense codons 11 times, sometimes with the same change occurring multiple times. All the nonsense mutations were GC-to-AT changes, which is the kind of mutation favored by the mutagen. Similarly, Couce et al. (2015) carried out experimental adaptation in *Escherichia coli* with replicate cultures from wild-type, mutH, and mutT parents, the latter two being “mutator” strains with elevated rates of mutation and different mutation spectra. When these strains were subjected to increasing concentrations of the antibiotic cefotaxime, the resulting adaptive changes reflected the differences in mutational spectrum between lines: resistant cultures from the mutT parent tended to adapt by a small set of A:T→C:G transversions, while resistant cultures from the mutH parent tended to adapt by another small set of G:C→A:T and A:T→G:C transitions.

Whereas these studies establish the empirical plausibility of mutational biases influencing the spectrum of parallel adaptation, the gap between highly manipulated laboratory studies and evolution in nature is large. Streisfeld and Rausher (2011) address the issue of the relative contributions of mutation bias and fixation bias to an evolutionary preference for regulatory versus structural changes. Though their main result was to find evidence of fixation bias, they also found an effect mutational bias. Recently, Galen et al. (2015) argued for the role of a CpG mutational hotspot in changes to hemoglobin linked to altitude adaptation in Andean house wrens (*Troglodytes aedon*; see also Stoltzfus and McCandlish 2015). However, as noted by Storz (2016), the importance of mutational biases in natural cases of parallel adaptation has not been investigated systematically.

Here we carry out a systematic analysis of published cases of parallel adaptation, focusing in particular on the influence of transition:transversion bias on parallel amino acid replacements. Sequence comparisons have long suggested a widespread mutational bias toward transitions, typically 2- to 4-fold over null expectations, with the lack of a bias being rare (Wakeley 1996; Keller et al. 2007). Direct studies of mutation rates confirm a transition bias, sometimes less than 2-fold over null expectations (e.g., Sung et al. 2012; Behringer and Hall 2015; Farlow et al. 2015) but more often in the range of 2- to 5-fold higher (e.g., de Boer and Glickman 1998; Keightley et al. 2009; Ossowski et al. 2010; Zhu et al. 2014; Foster et al. 2015; Dettman et al. 2016; Kucukyildirim et al. 2016; Smeds et al. 2016). Thus, if mutational biases substantially influence the dynamics of adaptation, we should see a strong enrichment of transition mutations among parallel adaptive substitutions.

Accordingly, we compiled data on experimental and natural amino acid replacements that have occurred two or more times, restricting ourselves to replacements where there is substantial evidence that the amino acid change is adaptive. These data include 63 replacements that arose independently during experimental adaptation a total of 389 times, and 55 replacements that occurred independently in nature a total of 231 times. Because for any wild-type nucleotide there are two possible transversions and only one possible transition, if mutational biases are irrelevant to adaptation then we should see approximately a 2-fold excess of transversions in our dataset. However, we instead observe that parallel transitions are as common or more common than transversions, consistent with the hypothesis that mutational biases play an important role in parallel adaptation.

## Results

We collected data from previously published studies providing examples of putatively adaptive amino acid changes that have occurred in at least two independent populations. We considered results of laboratory evolution separately from observations of an unsupervised process of adaptation that occurs outside of the laboratory. In molecular evolution, parallel changes may often occur without being adaptive (Natarajan et al. 2015; Thomas and Hahn 2015; Zou and Zhang 2015a, 2015b), therefore we never rely solely on sequence patterns as evidence for adaptation (Storz 2016). In the experimental cases, detected replacements are genetically linked to increases in fitness, and the chance that this linkage is non-causal is typically small; this is also true for a minority of the natural changes (13 of 55 paths), as when an insecticide-resistance phenotype maps to a locus with only a single replacement. For the remaining natural changes (42 of 55), the proposed functional effect is verified by a separate experimental result (typically via genetic engineering). Further details of these criteria are given in the Methods section.

In assembling and analyzing parallelism data, it is useful to distinguish the mutational *paths* by which parallel adaptation occurs from individual instances or *events* of change along a path. For instance, if we see a GTG valine at position 132 in a protein replaced with an ATG methionine independently in three different species, then this is one *path* (V132M) of parallel adaptation with three *events*. Because the frequency of transitions among paths is not necessarily the same as the frequency of transitions among events, we considered the frequency of transitions both among paths and among events.

As described in Methods, we compiled and verified information on parallels from natural and experimental studies until we obtained at least 50 paths of parallelism for each category.

### Formulation of Null and Alternative Models

Under the hypothesis that the category of a mutational change (transition or transversion) is irrelevant to the chances of being involved in adaptation, the observed frequency of transitions should be simply the frequency of transitions more generally among mutations that change proteins. We consider several models for what this frequency should be.

(1) If we assume that each single nucleotide mutation has an equal probability of being advantageous, the observed transition:transversion ratio will be 0.5, on the grounds that every nucleotide site is subject to one possible transition and two possible transversions.

(2) Given that our data include only replacements, a more precise expectation would exclude synonymous changes and calculate the expected transition:transversion ratio from amino acid replacements alone. Given the canonical genetic code, the 392 possible single-nucleotide mutations that change an amino acid consist of 116 transitions and 276 transversions, a ratio of 0.42.

(3) The previous calculation weights all non-synonymous mutations equally but one might consider the effects of codon usage. Using 12 different patterns of codon usage relevant to the species included in this study (see Methods), and weighting each possible non-synonymous mutation by the frequency of its ancestral codon, leads to a range of ratios from 0.40 to 0.42.

(4) Instead of the models above, one may consider a model of adaptation where, for a given ancestral codon, one of the amino acids accessible by a single nucleotide mutation becomes advantageous. This new amino acid then becomes fixed in the population by natural selection. There are a few cases in which the change from an ancestral codon to a mutationally accessible amino acid could occur by either a transition or a transversion, in which case we assume (conservatively) that only the transition paths are taken. Under these assumptions, we expect a slightly higher transition:transversion ratio of 0.49.

(5) Allowing for variation in codon usage in the above model produces a range of ratios from 0.48 to 0.50.

In summary, the expected transition:transversion ratio ranges between 0.4 and 0.5 depending on the details of the model. We therefore use the conservative estimate of 0.5 as our null expectation, representing the assumption that whether a mutation is a transition or a transversion is irrelevant to whether it is implicated in parallel adaptation.

Our alternative hypothesis in this study is that, because of the greater mutation rate towards transitions, these mutations will be overrepresented in cases of parallel adaptation, resulting in a ratio greater than 0.5. A ratio greater than 0.5 could also in principle be caused by a factor other than mutation, and in particular, if transition mutations tended to be more fit than transversion mutations, transitions might be overrepresented due to their fitness effects, rather than their elevated mutation rates. However, systematic fitness assays indicate that there is hardly any difference in fitness effects of transitions and transversions (Stoltzfus and Norris 2016; see also Dai, et al. 2016). We return to a variety of other alternative explanations for our observations in the Discussion.

### Experimental Parallelisms

To introduce the set of data aggregated from experimental cases (Table 1), we will begin by describing one case in detail, before briefly summarizing the other cases.

**Fig. 1.**
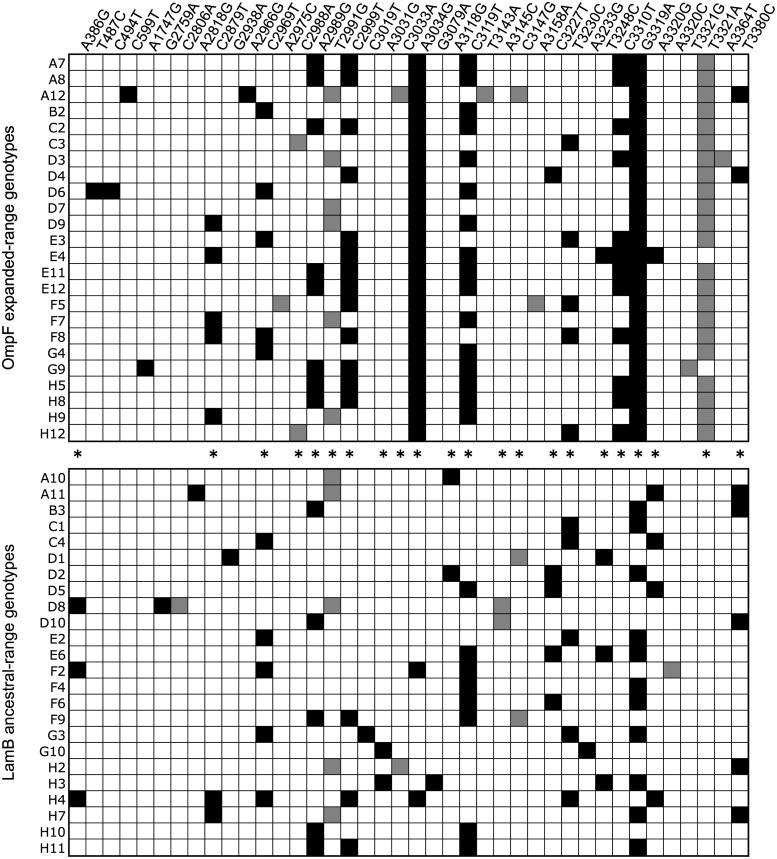
Parallel evolutionary changes (asterisks) in the *λ* tail tip gene (after fig. 1 of Meyer et al. 2012). Black (transition) and grey (transversion) boxes indicate specific nucleotide mutations (columns) found among 48 replicates (rows).

Meyer et al. (2012) propagated 96 replicate populations of bacteriophage *λ* on *E. coli* and monitored them periodically for the ability to grow on a LamB-negative host, indicating acquisition of an adaptive trait: the ability to utilize a second receptor (OmpF). Based on preliminary work establishing the J gene (encoding the tail tip protein) as a common locus of adaptation, they sequenced the J gene of 24 evolved strains with the ability to grow on a LamB-negative host, and a comparison set of 24 evolved strains without this ability. The complete set of 241 differences from the parental J gene in 48 replicates is shown in figure 1.

Meyer et al. (2012) did not experimentally verify each change. However, they report that all of the identified mutations were replacements, with no synonymous changes, which suggests that the frequency of hitch-hikers is low. The 95% confidence interval for the frequency of synonymous changes is 0 to 3/241=1.2%. If we assume that all hitch-hikers are synonymous, this leads to an upper-bound estimate on the frequency of hitch-hikers of 1.2%; if only half of hitch-hikers are synonymous, the value would be 2.4%. Either way, the chance of hitch-hiking is low, while the chance that a mutation identified by Meyer et al. (2012) is a driver is very high. Below (Discussion) we will estimate the level of contamination sufficient to influence our major conclusions and show that it is much larger than a few percent.

Among these non-synonymous mutations, 22 are found at least twice (asterisks in fig. 1), including 16 transitions found (collectively) 181 times, and 6 transversions found 42 times. In each case, these are exactly parallel nucleotide and amino acid changes. Thus the transition:transversion ratio is 16/6=2.7 when we count paths and 181/42=4.3 when we count events.

The other cases (described in more detail in Supplementary Material online) are as follows: Crill et al. (2000), extending the work of Bull et al. (1997), propagated a branching set of lines of φX174 through successive host reversals, switching between *E. coli* and *Salmonella typhimurium*, observing numerous reversals and 25 parallels; MacLean et al. (2010) adapted three starting genotypes of *Pseudomonas aeruginosa* to rifampicin, with 96 replicates each, and identified numerous rpoB mutations, 11 of which appeared in parallel; Rokyta et al. (2005) carried out 20 one-step adaptive walks with φX174 and carried out whole-genome sequencing, finding three parallelisms; Liao et al. (1986) selected seven temperature-resistant mutants of an *E. coli* kanamycin nucleotidyl-transferase expressed in *Bacillus stearothermophilus*, finding two parallelisms.

The data from all five experimental cases are summarized in table 1. Aggregating over all cases, the transition:transversion ratios are 43/20=2.2 (95% binomial confidence interval of 1.3 to 3.8) for paths and 304/85=3.6 for events (95% bootstrap confidence interval of 1.7 to 8.7 based on 10,000 bootstrap samples, see Methods). These ratios are 4-fold and 7-fold (respectively) higher than the conservative null expectation of 0.5. The excess of transition paths is highly significant by a binomial test ($P<10^{-5}$; here and below, we do not distinguish values of *P* below $10^{-5}$). By randomly reassigning mutations to be transitions or transversions under the assumption that the null hypothesis is true, we can test separately for a significant enrichment of transition events over the null ratio of 0.5 (see Methods). By such a test, the observed bias in events is highly significant ($P<10^{-5}$, based on 10^6^ randomizations).

**Table 1.** Summary of Paths and Events for Each of the Five Experimental Cases.

			Paths		Ti Events		Tv Events	
Phenotype	Taxon	Target	Ti	Tv	Counts	Sum	Counts	Sum
High-T host adaptation	*φ*X174	genome	17	8	4, 3, 2, 2, 4, 2, 2, 3, 4, 4, 2, 2, 4, 2, 4, 3, 2	49	2, 3, 2, 4, 3, 3, 2, 4	23
Host adaptation	Lambda	J protein	16	6	3, 7, 10, 13, 16, 2, 26, 2, 24, 5, 10, 4, 12, 35, 5, 7	181	2, 11, 2, 2, 3, 22	42
Rifampicin resistance	*P. aeruginosa*	RNAPol	7	4	4, 35, 2, 5, 2, 4, 9	61	3, 3, 2, 3	11
Increased fitness	*φ*X174	genome	3	0	5, 2, 6	13		0
Kanamycin resistance	*Escherichia coli*	KNTase	0	2		0	7, 2	9
Total			43	20		304		85

Table 2 shows the result of restricting our analysis to events that have occurred in parallel *k* or more times for *k* = 2 through 8. Increasingly stringent criteria for inclusion should result in a decreased frequency of hitch-hikers and other neutral contaminants. However, our results remain qualitatively similar and highly significant even for these much smaller subsets of data.

**Table 2.** Results from Experimental Cases under Increasing Cutoffs for the Minimum Number of Parallel Events per Path.

	Paths				Events			
Cutoff	Ti	Tv	Ratio	*P*-value	Ti	Tv	Ratio	*P*-value
2	43	20	2.2	<1 × 10^−5^	304	85	3.6	<1 × 10^−5^
3	30	12	2.5	<1 × 10^−5^	278	69	4.0	<1 × 10^−5^
4	26	5	5.2	<1 × 10^−5^	266	48	5.5	<1 × 10^−5^
5	17	3	5.7	<1 × 10^−5^	230	40	5.8	1.7 × 10^−5^
6	13	3	4.3	1.16 × 10^−4^	210	40	5.3	1.41 × 10^−4^
7	12	3	4.0	2.85 × 10^−4^	204	40	5.1	2.32 × 10^−4^
8	10	2	5.0	5.44 × 10^−4^	190	33	5.8	4.38 × 10^−4^

### Natural Parallelisms

We will begin by considering a case that illustrates most of the complexities of natural cases, and then present a brief description of the other cases (Table 3). The Supplementary Material online provides a more complete description with evidence pertaining to each path. Note that, while an experimental case (above) usually corresponds to a single published study with results from one laboratory, a natural case more typically draws on multiple published studies or reviews that cover the same system.

Some naturally occurring glycoside toxins target the sodium pump ATP*α*1. These include cardenolides produced by milkweed and other members of the dogbane family (*Apocynaceae*), as well as cardiac glycosides produced by amphibians. Some insects have resistance allowing them to eat *Apocynaceae*; species such as monarch butterflies (*Danaus plexippus*) not only consume the toxin but sequester it so as to make themselves noxious to predators. Resistant insects typically have undergone changes in ATP*α*1: the effects of many specific mutations have been explored via genetic engineering followed by functional and structural analysis.

**Fig. 2.**
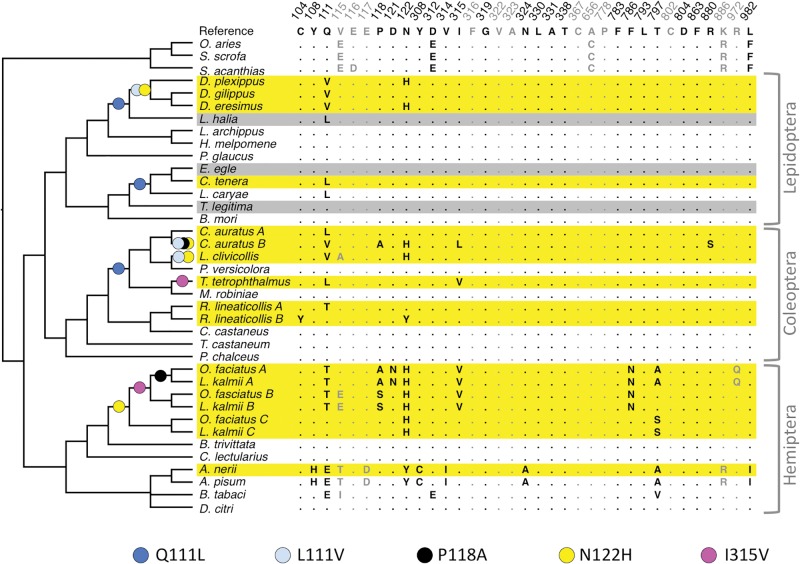
Parallel changes in ATP*α*1 in various insect species that sequester (yellow) or merely consume (grey) cardenolides. The only ATP*α*1 sites (columns) shown here are those implicated in cardenolide-binding by experimental mutations (black font) or structural modeling (grey font). Colored dots show inferred evolutionary replacements that are counted as adaptive parallels (see text).

The entire set of data on ATP*α*1 parallelisms reported by Zhen et al. (2012) is illustrated in figure 2, based on figure 1 of Zhen et al. (2012). Yellow-shaded species consume and sequester plants producing cardenolides; grey-shaded species merely consume them. Figure S1 of Zhen et al. (2012) summarizes the sources of information on functional effects of replacements. For instance, Croyle *et al.* (1997) carried out random mutagenesis and screening of ATP*α*1 mutants for ouabain resistance, with results implicating the T797A replacement as well as replacements at sites 111, 118, and 122.

From figure 2, the replacements P118A (black), N122Y I315V (magenta), and T797A clearly happen twice each. The most parsimonious reconstruction for site 122 would call for one change to H in Hemiptera, two changes in Coleoptera, and either two changes to H, or one change plus a reversal in Lepidoptera. We count this conservatively as four changes. Site 111 illustrates unusual complexity. Q111L (blue) is a single transversion CAR→CTR that occurs several times. Q111V implicates two changes, most parsimoniously (as argued in Aardema et al. 2012) derived from a Q111L ancestor, that is, Q→L→V via CAR→CTR→GTR. Thus, the pattern in the minimal clade containing *D. plexippus* and *Lycorea halia* indicates Q→L (blue) in the ancestral lineage and then L→V (light blue) in the *Danaus* ancestor. Severally equally parsimonious scenarios involve five changes at site 111 in the clade that includes *Chrysochus auratus* and *Megacyllene robiniae*; the scenario with the fewest parallels is shown (this entails two L→Q reversals in *M. robiniae* and *Plagiodera versicolora* that we do not count). Q111T, another double-nucleotide change, happens twice with no evidence of intermediates: even if one could assume that both changes occurred via successive single-nucleotide replacements, the path is ambiguous (Q→P→T or Q→K→T), therefore no parallel can be inferred.

Zhen et al. (2012) do not count all of these as verified adaptive parallels. Though T797A has been verified experimentally, the occurrence of 797A and 122Y in the aphid clade has no strong correlation with cardenolide consumption, as there is one consumer (*A. nerii*) and one non-consumer (*A. pisum*). Thus, though these are parallels, we follow the original authors in not counting them as genuine adaptive parallels consistent with the hypothesis that associates cardenolide utilization with resistance via changes in ATP*α*1.

Using *ti* and *tv* to represent transitions and transversions, the parallel changes are Q111L CAR→CTR (3 tv), L111V CTR→GTR (2 tv), P118A CCN→GCN (2 tv), N122H AAY→CAY (4 tv), and I315V ATH→GTH (2 ti). All five replacements are implicated functionally by experimental results summarized in the Supplementary Material online.

Ujvari et al. (2015) report some additional parallels among mammals and squamates (lizards and snakes) that consume glycoside-bearing plants, insects, or toads. The set of replacements shown in their figure 2 (see also their supplementary fig. S1) includes the Q111L and N122H events in insects indicated above, four additional events of Q111L, and five other paths. One of the paths (Q111E) is seen only once, and another path with two events (Q119D) is a double-nucleotide change, thus not counted here. The G120R change that occurs four times in squamates is ambiguously either GGR→AGR (ti) or GGN→CGN (tv): inspecting columns 70 to 72 of the aligned sequences from Ujvari et al. (2015) (NCBI popset 928240786) indicates that the change in all four cases is GGA→AGA (ti). The remaining paths observed are Q111R CAR→CGR (4 ti) and N122D AAY→GAY (2 ti).

The aggregated data set for natural evolution consists of the case of glycoside-resistance, along with nine other cases, as summarized in table 3. The cases in which the detection of parallels is targeted at prestin (Liu et al. 2014), opsins (Shyue et al. 1995; Yokoyama and Radlwimmer 2001), hemoglobin (McCracken et al. 2009; Projecto-Garcia et al. 2013; Natarajan et al. 2015, 2016), and ribonuclease (Zhang 2006; Yu et al. 2010) involve well-known phenotypic parallelisms for echolocation, trichromatic vision, altitude adaptation, and foregut fermentation, respectively. The cases of resistance to glycosides (Zhen et al. 2012; Ujvari et al. 2015) and tetrodotoxin (Jost et al. 2008; Feldman et al. 2012) involve a naturally evolved response to a natural toxin. The cases of resistance to insecticides (Weill et al. 2003; ffrench-Constant et al. 2004; Soderlund 2005), benzimidazole (Koenraadt et al. 1992; Elard et al. 1996), herbicides (Liu et al. 2007), and ritonavir (Molla et al. 1996) involve the unsupervised natural evolution of resistance to human-produced toxins.

**Table 3.** Summary of Paths and Events for Each of the Ten Natural Cases.

			Paths		Ti Events		Tv Events	
Phenotype	Taxon	Target	Ti	Tv	Counts	Sum	Counts	Sum
Insecticide resistance	Insecta	Rdl, Kdr, Ace	5	3	2, 2, 5, 2, 3	14	9, 2, 4	15
Tetrodotoxin resistance	Vertebrata	Na channels	3	5	2, 6, 3	11	2, 2, 2, 3, 3	12
Glycoside resistance	Metazoa	*Na*^+^/*K*^+^-ATPase	4	4	4, 4, 2, 2	12	7, 2, 2, 4	15
Herbicide resistance	Poaceae	ACCase	2	4	5, 2	7	7, 2, 4, 5	18
Altitude adaptation	Aves	*β*-hemoglobin	2	3	4, 13	17	2, 3, 2	7
Trichromatic vision	Vertebrata	Opsins	2	3	2, 5	7	6, 4, 2	12
Echolocation	Mammalia	Prestin	3	2	2, 2, 2	6	3, 2	5
Growth in Ritonavir	HIV1	Protease	3	1	25, 7, 9	41	4	4
Foregut fermentation	Vertebrata	Ribonucleases	3	0	2, 4, 4	10		0
Benzimidazole resistance	Ascomycota	*β*-tubulin	1	2	7	7	5, 6	11
Total			28	27		132		99

Out of 55 paths, we expect 18.3 transition paths under the null model. However, the observed number of transition paths is 28, corresponding to a transition:transversion ratio of 1.0 (95% binomial confidence interval from 0.62 to 1.8), which is 2-fold higher than the null expectation ($P=5.3\times 10^{-3}$ by a binomial test). Similarly, out of the 231 parallel events, 132 of them are transitions, corresponding to a transition:transversion ratio of 1.3 (95% bootstrap confidence interval of 0.66 to 2.6 based on 10,000 bootstrap samples, see Methods). This is 2.5-fold higher than would be expected under our null model ($P=3.0\times 10^{-3}$, based on 10^6^ randomizations, see Methods).

Table 4 shows the results of restricting our analysis to paths that have been observed at least *k* times for *k* = 2 to 8. The results remain qualitatively unchanged even under higher-stringency cutoffs that should eliminate most non-adaptive contaminants.

**Table 4.** Results from Natural Cases under Increasing Cutoffs for the Minimum Number of Parallel Events per Path.

	Paths				Events			
Cutoff	Ti	Tv	Ratio	*P*-value	Ti	Tv	Ratio	*P*-value
2	28	27	1.0	5.28 × 10^−3^	132	99	1.3	3.05 × 10^−3^
3	16	16	1.0	3.77 × 10^−2^	108	77	1.4	9.28 × 10^−3^
4	14	12	1.2	2.48 × 10^−2^	102	65	1.6	8.5 × 10^−3^
5	9	7	1.3	5 × 10^−2^	82	45	1.8	1.63 × 10^−2^
6	6	5	1.2	0.12	67	35	1.9	3.58 × 10^−2^
7	5	3	1.7	8.79 × 10^−2^	61	23	2.7	3.49 × 10^−2^
8	3	1	3.0	0.11	47	9	5.2	6.18 × 10^−2^

## Discussion

To explore the role of mutational biases in parallel adaptation, we gathered data from published studies in which adaptation can be linked to nucleotide mutations that cause amino acid replacements, either in nature or in the lab. We used these data to test for an effect of transition:transversion bias, a widespread kind of mutation bias. In the experimental data set of 389 parallel events along 63 paths, we find a highly significant tendency—from 4-fold to 7-fold in excess of null expectations—for adaptive changes to occur by transition mutations rather than transversion mutations. For the dataset of natural cases of parallel adaptation consisting of 231 parallel events along 55 paths, we found a bias of 2-fold to 3-fold over null expectations, which was statistically significant for both paths and events. Parallel adaptation appears to take place by nucleotide substitutions that are favored by mutation, and the size of this effect is not a small shift, but a substantial effect of 2-fold or more.

While we have focused on cases of parallel adaptation, our results have implications for understanding the roles of mutation and selection more generally. Historically, mutation and selection have often been cast as opposing forces (Fisher 1930; Arthur 2001; Yampolsky and Stoltzfus 2001). Indeed, when considering a single bi-allelic locus, the dynamics are essentially one-dimensional, so that mutation and selection must act in either the same or opposite directions, with selection typically dominating the outcome in either circumstance. However, in the vastness of sequence space, mutational and selective biases may act more like vectors pointing in different—rather than opposite—directions (Stoltzfus and Yampolsky 2009; Stoltzfus 2012). How these vectors combine, and the resultant course of adaptation, depends on the population-genetic details. Contemporary theory suggests that mutational biases will have little effect in panmictic populations with abundant segregating variation, but a strong effect in mutation-limited populations, whether due to small population size (Yampolsky and Stoltzfus 2001; McCandlish and Stoltzfus 2014) or spatial structure (Ralph and Coop 2015). Thus, detecting a substantial influence of mutational biases suggests that adaptation in both laboratory and natural populations is to some extent mutation limited.

Our conclusions concerning the role of transition:transversion bias in parallel adaptation follow if the observed excess of transitions indeed reflects mutation bias rather than a different mechanism that would also enrich for transitions. Are there other ways to account for this excess? One possibility is that transitions are systematically fitter than transversions. As noted earlier, laboratory studies do not show a substantial fitness advantage of transitions over transversions (Dai et al. 2016; Stoltzfus and Norris 2016). However, these studies examine the entire distribution of fitness, and do not have the power to resolve differences far in the right tail of rare beneficial mutations. That is, transitions might be favored among beneficial mutations even if they are not favored overall, and this could explain the observed excess of transitions among parallel adaptive changes.

Other alternative explanations could be based on contamination of the data by changes that are not adaptive parallels and are biased toward transitions. Given that molecular changes in general are biased toward transitions (Wakeley 1996), various scenarios of contamination by hitch-hikers or misidentification of changes would impose a bias toward transitions. For the experimental cases, we have prior reasons to believe that the vast majority of reported parallel mutations are drivers. Furthermore, the observed 43:20 excess of transitions is so extreme that, if we consider the contamination hypothesis assuming (for the sake of argument) that all contaminants are transitions, the data would need to consist of 30 paths that follow the null distribution (10 transitions and 20 transversions), along with 33 contaminant paths. That is, to explain the observed excess under the contamination hypothesis is to propose that most of the data are contaminants.

Although our confidence is strong for the laboratory cases, more doubt exists for the natural cases, because the excess of transitions is smaller, and the prior probability for contamination is greater. Some putative instances of natural adaptive amino acid parallelisms in the literature are now believed to be non-adaptive or non-independent (Natarajan et al. 2015; Aardema and Andolfatto 2016). More generally, the appearance of parallel amino acid changes could be due to adaptive introgression (Mallet et al. 2016), or incomplete lineage sorting (Mendes and Hahn 2016). However, if we again assume conservatively that all misidentifications and contaminants are transitions, explaining the observed 28:27 ratio would require 25% contamination—14.5 contaminants mixed with 40.5 genuine parallels that exhibit the null 13.5:27 ratio—, which seems unlikely given that each is accompanied by specific evidence in the form of genetic association or experimental validation.

Though an enrichment for transitions was found in both natural and experimental cases, it was considerably stronger for the experimental cases. Broadly speaking, the experimental and natural cases may differ systematically in many ways, including mutation rates and biases, extent of recombination, degree of environmental heterogeneity, population size, strength of selection, and duration of selection. It is therefore difficult to assess which of these differences is responsible for the observed difference in transition:transversion ratio. Moreover, we caution that in our compilation of data, the natural-experimental distinction is confounded with a taxonomic distinction, in that the experimental cases involve only eubacteria and their phages, whereas the natural cases involve primarily multicellular eukaryotes.

Another pattern in our data is the greater transition:transversion ratio among events than paths. This phenomenon can be understood in terms of the differing effects of increasing the number of replicate populations at the level of paths versus the level of events. With very few replicate populations, the transition:tranversion ratio will be the same for events and paths because adaptive replacements will be seen at most twice, and so each parallel path will contribute exactly two parallel events. However, as the number of replicate populations increases each *possible* beneficial path will be observed multiple times. Because all possible beneficial substitutions will be observed as parallelisms, the transition:transversion ratio at the level of events will converge on the transition:transversion ratio for adaptive replacements more generally, and will show an enrichment for transitions under the hypothesis that adaptive evolution is influenced by mutational biases. However, at the level of paths, the transition:transversion ratio will converge to the ratio of the number of possible beneficial transitions versus the number of possible beneficial transversions, which we argued earlier should be approximately 0.5. As a result, we expect that when there are many replicate populations, the transition:tranversion ratio among events should be higher than that among paths.

Here we have shown that a specific type of mutational bias, transition-transversion bias, is strongly reflected in the distribution of changes during parallel adaptation. Recent anecdotal evidence from Galen et al. (2015) suggests that a similar effect may occur due to elevated mutation rates at CpG sites, while Bailey et al. (2017) present a meta-analysis of paired mutation-accumulation and evolve-and-resequence studies showing an effect of mutational target size and gene-specific mutation rate for the distribution of parallel changes. Other biases in the mutational spectrum such as insertions versus deletions, AT-to-GC bias, and context-dependent mutation are also worthy of investigation. The extent to which these other biases shape the distribution of adaptive changes remains an open question.

## Material and Methods

### Cases

Candidate cases of experimental and natural adaptation were identified from available reviews of parallel evolution or experimental adaptation (Wood et al. 2005; Arendt and Reznick 2008; Gompel and Prud’homme 2009; Stern and Orgogozo 2009; Christin et al. 2010; Conte et al. 2012; Martin and Orgogozo 2013; Stern 2013; Stoltzfus and McCandlish 2015; Long et al. 2015; Lenormand et al. 2016). We processed cited works opportunistically for each set until we had accumulated cases comprising at least 50 parallelisms. For the natural cases, the inclusion of two additional sources in response to a reviewer’s request led to the addition of four paths and 17 events. The resulting data sets include 55 and 63 paths for the natural and experimental cases, respectively.

Processing of a case may involve finding newer and more complete reviews, combining data from multiple publications on the same system (and thus resolving duplications and conflicting numbering schemes), reviewing claims attributed to cited publications, and checking GenBank sequences to infer mutations from the identities of codons (which are often not reported). We did not carry out new synthetic work such as BLAST searches or literature searches in order to expand the scope of published cases, but rather took the approach of a meta-analysis in which the scope of a case is defined by what experts have chosen to include in published works.

We use only studies that (1) implicate exactly parallel amino acid changes where (2) the changes evidently represent new mutations and not shared ancestral variation, and (3) there is evidence beyond the mere pattern of parallelism suggesting that the mutation is a driver rather than a non-driver.

For experimental studies of adaptation, the evolved organism exhibits an increase in growth, or an increased ability to survive a threat (e.g., a toxin or pathogen). Detected replacements are thus linked with a measured effect, and sometimes this linkage implicates a single replacement. Even in a case such as Meyer et al. (2012), where many isolates have multiple replacements, the level of contamination by hitch-hikers is likely to be low as evidenced by the low frequency of synonymous changes (see main text).

For reported natural parallelisms, to reduce the chance of spurious parallels, we do not include any paths identified merely from a phylogenetic pattern of recurrence, even if the changes occur in a candidate gene and appear to be significant by some kind of statistical model, as in Zhang and Kumar (1997). For a minority of paths (13 of 55), there is a genetic association with a phenotype, the nature of which is typically the same type as in the experimental cases: there is an evolved difference in a measured quantity such as toxin resistance, and the comparison of sequences linked to the difference implicates a replacement specifically, often because it is the only one. For the remaining paths (42 of 55), there is an experimental result—separate from the original observation of an evolved replacement in a particular context—that links the replacement to a functional effect consistent with the adaptive hypothesis. Typically this experiment involves site-directed mutagenesis but there are also cases in which selection following unsupervised mutagenesis reveals which replacements have relevant functional effects. The Supplementary Material online describes, for each natural path, the evidence used to make this determination.

In most cases, the identity of a nucleotide mutation can be deduced from the amino acid change, for example, a change from Pro to Ser is always a C→T transition in the first position of a CCN codon. In ambiguous cases, we consult sequences and count two events as the same if they implicate the same codon position (1, 2, or 3) from the same ancestral block of synonymous codons, and are either both transitions or both transversions.

### Collation of Data

Data from studies identified as described above were processed manually. In a typical experimental case, a table of results must be extracted from a published paper and analyzed in an ad hoc manner to identify parallels. In a typical natural case, a figure illustrating character states in the context of a phylogeny must be interpreted manually, following the authors’ interpretation and applying the rules of parsimony. To reduce the impact of errors in interpretation and of clerical errors, each study was analyzed in duplicate. We observed slight changes between the replicates that did not alter the conclusions of an analysis; these issues were then resolved to construct the final dataset. Thus, although we cannot guarantee that the results presented here are completely free from clerical errors and ambiguities in interpretation, we believe that such uncertainties do not effect the conclusions.

Codon usage data for *Arabidopsis thaliana*, *B. subtilis*, *Coprinopsis cinerea*, *Gallus gallus*, *E. coli*, *Drosophila melanogaster*, *HIV*, *Homo sapiens*, *bacteriophage Lambda*, *bacteriophage phiX174*, *Oryza sativa*, and *Saccharomyces cerevisiae* were downloaded from the CUTG database (Nakamura et al. 1998).

### Statistical Tests

Statistical tests were conducted in Mathematica, and a Mathematica notebook implementing these tests is included with the Supplementary Material online. The statistical analysis for paths consisted of binomial tests and binomial confidence intervals. For events, we could not conduct an exact analysis because of the variable number of events per observed path, therefore we resorted to Monte Carlo techniques. Confidence intervals for events were calculated using 10,000 bootstrap samples. More specifically, for each bootstrap sample we resampled with replacement from the set of observed paths, determined the total number of transition events and total number of transversion events, then calculated the transition:transversion ratio. Hypothesis tests for events were conducted by randomly reassigning paths to be either transitions or transversions based on a transition:transversion ratio of 0.5. This was repeated 10^6^ times to construct the sampling distribution of the transition:transversion ratio under the null. Reported *P*-values are one-sided, based on our a priori hypothesis that the transition:transversion ratio should be elevated among parallel replacements.

## Supplementary Material

Supplementary data are available at *Molecular Biology and Evolution* online.

## Supplementary Material

Supplementary Data
