# Supplementary material for: Mutational Biases Influence Parallel Adaptation
Source: Mol Biol Evol. 2017 Jun 22;34(9):2163–72. doi: 10.1093/molbev/msx180 (PMC5850294; doi:10.1093/molbev/msx180)
Supplement: Supplementary Data [file msx180_Supp.zip › SupplementaryText.pdf]

Supplementary text for:

Mutational biases influence parallel adaptation

Arlin Stoltzfus[1] and David M. McCandlish[2]

[1]Email: arlin@umd.edu; Corresponding author
[2]Email: mccandlish@cshl.edu

Narrative descriptions of cases, results, and evidence

The supplementary material consists of a narrative description of each case, including a detailed account listing every parallel change.

Experimental cases

Tip attachment protein: parallel host-specificity changes (phage λ). Meyer *et al.* (2012) propagated 96 replicate populations of bacteriophage λ on *E. coli*, and monitored them periodically for the ability to grow on a LamB-negative host, indicating evolution of a new OmpF receptor capability. Based on preliminary work establishing the J gene as a common locus of adaptation, they sequenced the J gene of 24 evolved strains with the ability to grow on a LamB-negative host, and a comparison set of 24 evolved strains without this ability. The authors report that all 241 detected changes from the parental J gene sequence are non-synonymous. From among this set, the complete set of parallel differences in 48 replicates is as follows:

- 3 events of gene J site 386 $A \to G$ (ti)
- 7 events of gene J site 2879 $C \to T$ (ti)
- 10 events of gene J site 2969 $C \to T$ (ti)
- 2 events of gene J site 2988 $C \to A$ (tv)
- 13 events of gene J site 2989 $A \to G$ (ti)
- 11 events of gene J site 2991 $T \to G$ (tv)
- 16 events of gene J site 2999 $C \to T$ (ti)
- 2 events of gene J site 3031 $A \to G$ (ti)
- 2 events of gene J site 3033 $C \to A$ (tv)
- 26 events of gene J site 3034 $A \to G$ (ti)
- 2 events of gene J site 3118 $A \to G$ (ti)
- 24 events of gene J site 3119 $C \to T$ (ti)
- 2 events of gene J site 3145 $A \to C$ (tv)
- 3 events of gene J site 3147 $C \to G$ (tv)
- 5 events of gene J site 3227 $C \to T$ (ti)
- 10 events of gene J site 3230 $T \to C$ (ti)
- 4 events of gene J site 3248 $T \to C$ (ti)
- 12 events of gene J site 3310 $C \to T$ (ti)
- 35 events of gene J site 3319 $G \to A$ (ti)
- 5 events of gene J site 3320 $A \to G$ (ti)
- 22 events of gene J site 3321 $T \to A$ (tv)
- 7 events of gene J site 3380 $T \to C$ (ti)

Genomic changes in adaptation to host reversals (ϕX174). Bull *et al.* (1997) and later Crill *et al.* (2000) studied ϕX174 evolution under successive reversals of the host species. The experimental protocol of Bull *et al.* (1997) can be expressed with the Newick string

$(C1:11,(C2+:22)C2:11,(SC1:22,SC2:11,S1+:22)S1:11,S2:11,S3:11)phiX174\_strainA$, where the branch lengths are days in continuous culture at elevated temperature (42 C), and the "C" and "S" letters indicate whether the host is *Escherichia coli* (C) or *Salmonella typhimurium* (S). The description of methods on p. 1498 (left column) indicates that transfers from one branch to the next are clonal isolates used for sequencing. This is an important detail because it means that population variation is not carried over from one branch to the next: all apparent reversions and parallels are from new mutations. Efforts were made to avoid cross-contamination of cultures; most cultures were propagated in Texas, while one was propagated in Idaho.

Crill *et al.* (2000) extended the original study by taking a culture subjected to host reversal in the original study and subjecting it in duplicate to two more host reversals. That is, an SC culture was subjected in duplicate to SCS and then SCSC. Though the authors were interested in reversals rather than parallels, their results included many parallel changes, in the sense that the same change occurred in different lineages, or in the same lineage following a reversion. Table 2 of Crill *et al.* (2000) indicates the changes that involve reversion, including ones reported previously by Bull *et al.* (1997). Note that the focus of the authors is on reversions, therefore the table may be missing some changes that are parallels but not reversions. How the successive rows of data in Table 2 of Crill *et al.* (2000) affect the tally of parallel replacements from Bull *et al.* (1997) is as follows:

- for gene C site 319, no new parallel
- for gene D site 500, synonymous, not counted
- for gene J site 921, new reversion but no parallel
- for gene F site 1305 , 2 more $G \rightarrow A$ forward changes (SCS1, SCS2), and 2 more $A \rightarrow$G reversions (SCSC1, SCSC2)
- for gene F site 1460, new reversion but no parallel
- for gene F site 2009, 1 more $T \rightarrow A$ forward change (SCS2), 1 more $A \rightarrow T$ reversion (SCSC2)
- for gene F site 2093, new reversion but no parallels
- for gene F site 2167, no new parallel
- for gene A site 4420, no new parallel
- for gene A site 4700, no new parallel
- for gene A site 4805, synonymous, not counted

Thus, Crill *et al.* (2000) adds forward and reverse changes at only two sites. The combination of the two studies is then as follows:

- 4 events of gene A site 4110 $C \rightarrow T$ (ti) in C2+, S1, S2, S3
- 3 events of gene A site 4122 $G \rightarrow A$ (ti) in C1, C2, S1
- 2 events of gene A site 4168 $A \rightarrow G$ (ti) in C2+, S1
- 2 events of gene A site 4420 $A \rightarrow C$ (tv) in S1, S3
- 2 events of gene B site 31 $A \rightarrow G$ (ti) in C1, SC1
- 3 events of gene C site 319 $G \rightarrow T$ (tv) in C1, S1, S3
- 4 events of gene D site 756 $T \rightarrow C$ (ti) in C1, C2, S1, S2

- 2 events of gene D site 795 $A \to G$ (ti) in SC1, SC2
- 2 events of gene J site 905 $G \to A$ (ti) in SC1, S2
- 3 events of gene F site 1025 $G \to A$ (ti) in C1, S1, S3
- 4 events of gene F site 1305 $G \to A$ (ti) in S1, S2, SCS1, SCS2
- 4 events of gene F site 1305 $A \to G$ (ti) in SC1, SC2, SCSC1, SCSC2 (reversion)
- 2 events of gene F site 1533 $C \to T$ (ti) in C2, S2
- 2 events of gene F site 1565 $A \to G$ (ti) in SC1, S1+
- 2 events of gene F site 1613 $A \to T$ (tv) in S1, S3
- 4 events of gene F site 1727 $C \to T$ (ti) in C1, C2, SC2, S2
- 4 events of gene F site 2009 $T \to A$ (tv) in S1, S2, S3, SCS2
- 3 events of gene F site 2009 $A \to T$ (tv) in SC1, SC2, SCSC2 (reversion)
- 3 events of gene F site 2167 $T \to G$ (tv) in S1, S2, S3
- 2 events of gene F site 2167 $G \to T$ (tv) in SC1 and SC2 (reversion)
- 2 events of gene G site 2591 $T \to C$ (ti) in C1, SC2
- 4 events of gene H site 2980 $C \to T$ (ti) in C1, C2, SC2, S2
- 3 events of gene H site 3120 $C \to T$ (ti) in C2, SC2, S2
- 2 events of gene H site 3337 $C \to T$ (ti) in SC1, SC2
- 4 events of gene H site 3378 $A \to C$ (tv) in C1, C2, SC1, SC2

RNA polymerase: acquiring rifampicin resistance (*Pseudomonas aeruginosa*). MacLean *et al.* (2010) adapted 96 replicate cultures of *Pseudomonas aeruginosa* to increasing concentrations of rifampicin. Prior work establishes that resistance is usually due to changes in a region of rpoB sequenced by the authors. Sequencing revealed 36 mutations, including 35 amino acid replacements and 1 insertion. The parallel paths and their numbers of occurrences can be derived from the frequency data provided in their Table 1, by inferring 70, 78 and 78 as the denominators of frequency in the three backgrounds. The results are as follows:

- 3 events of site 1549 $T \to G$ (tv)
- 4 events of site 1553 $A \to G$ (ti)
- 3 events of site 1553 $A \to T$ (tv)
- 35 events of site 1562 $A \to G$ (ti)
- 2 events of site 1563 $C \to G$ (tv)
- 2 events of site 1567 $A \to G$ (ti)
- 5 events of site 1591 $C \to T$ (ti)
- 3 events of site 1591 $C \to G$ (tv)
- 2 events of site 1607 $C \to T$ (ti)
- 4 events of site 1736 $C \to T$ (ti)
- 9 events of site 449 $T \to C$ (ti)

Genomic changes during one-step adaptive walks (bacteriophage ϕX174). Rokyta *et al.* (2005) carried out 20 one-step adaptive walks (i.e., the experiment is ended when a fitness

increase is detected) with laboratory populations of bacteriophage ϕX174, finding 9 different amino acid replacements. Here we consider only the recurrent replacements, which occurred 5, 3 and 6 times. The $Pro \rightarrow Ser$ change at position F355 is a $CCN \rightarrow TCN$ transition, so we count this as 5 transitions. The $Met \rightarrow Ile$ change that occurred 3 times at position 416 of the F gene might be either $ATG \rightarrow ATA$ (ti) or $ATG \rightarrow ATY$ (tv). The actual results involved 2 transitions and 1 transversion (Darin Rokyta, personal communication). The $Ala \rightarrow Val$ change that occurred 6 times at position J15 is a $GCN \rightarrow GTN$ transition. The results may be summarized succinctly as follows:

- 5 events of P(F355)S $CCN \rightarrow TCN$ (ti)
- 2 events of M(F416)I $ATG \rightarrow ATA$ (ti)
- 6 events of A(J15)V $GCN \rightarrow GTN$ (ti)

Nucleotidyl transferase: kanamycin resistance (*Bacillus stearothermophilus*). Liao *et al.* (1986) repeatedly selected for resistance to kanamycin at high temperatures in a thermophilic organism, *Bacillus stearothermophilus*, using a cloned kanamycin nucleotidyltransferase from *Escherichia coli*. They found that 7 different isolates all had the D80Y change, and 2 of them also underwent a T130K change.

- 7 events of D80Y $GAT \rightarrow TAT$ (tv)
- 2 events of T130K $ACG \rightarrow AAG$ (tv)

Natural cases

Sodium channels: resistance to tetrodotoxin (vertebrates). Tetrodotoxin (TTX) is a potent natural toxin used for defense in pufferfish, many amphibians, and invertebrates from a handful of phyla (see references in Jost *et al.* 2008). Resistance in snakes that prey on amphibians has arisen at least 6 times (Feldman *et al.*, 2012). The interaction of TTX with voltage-gated sodium channels has been studied for decades, and much is known about the effects of replacements. Jost *et al.* (2008) use this information, as well as their own site-directed mutagenesis experiments, to identify functionally important changes in paralogous families of sodium channels in 4 species of pufferfish. Most of the changes occur in or near the exposed loops of subunits denoted Domain I to Domain IV.

The ones that occur in parallel in pufferfish and in other cases from the literature collated by Jost *et al.* (2008) are 2 events each of Y(DI.p2)C, E(DII.p4)D, and T(DII.p5)S, 6 events of M(DIII.p4)T, 3 events of A1529G, 3 events of I1561M, and 2 events of D(DIV.p4)N. Here "DII.p2" means "domain II, pore loop position 2". Note that the authors over-count A1529G, by including a change in the Nav1.1La sub-family that is found in the *Danio rerio* outgroup, thus not restricted to TTX-resistant pufferfish.

For Y(DI.p2)C, E(DII.p4)D, T(DII.p5)S, I1561M and D(DIV.p4)N, the authors cite earlier studies indicating that either the specific replacement, or a chemically similar replacement, is implicated in TTX resistance. For the M(DIII.p4)T change, the authors used site-directed mutagenesis to introduce this change to a rat sodium channel (from skeletal muscle), finding a 15-fold increase in TTX-resistance. For the A1529G change, they used site-directed mutagenesis of a mammalian Nav1.4 channel to show a modest 1.5-fold effect on TTX resistance, along with an apparent increase in channel conductance.

I1561M is ambiguous, being either an $ATA \rightarrow ATG$ transition, or an $ATY \rightarrow ATG$ transversion. We score this as a transversion because related sequences indicate an ATC codon at this site (e.g., XM_010856557.1 site 5619, JQ687861.1 site 191, and XM_017710591.1 site 4611).

These results are extended slightly by Feldman *et al.* (2012), who identify 2 exact parallels among snakes that prey on tetrodotoxin-bearing amphibians, D(DIII.p5)E (2 tv) (site 1277) and D(DIV.p4)N (2 ti) (site 1568). Both Jost *et al.* (2008) and Feldman *et al.* (2012) cite the same D(DIV.p4)N change in *Thamnophis sirtis*, thus the total from both studies is 3 rather than 4. Feldman *et al.* (2012) also find another instance of the M(DIII.p4)T change identified by Jost *et al.* (2008). Thus, the total from the two studies is as follows:

- 2 events of Y(DI.p2)C, $TAY \rightarrow TGY$ (ti), from Jost *et al.* (2008): this replacement is implicated in reduced toxin affinity by a mutation engineered by Venkatesh *et al.* (2005).
- 2 events of E(DII.p4)D, $GAR \rightarrow GAY$ (tv), from Jost *et al.* (2008): this replacement is implicated in toxin resistance by a mutation engineered by Bricelj *et al.* (2005).
- 2 events of T(DII.p5)S, $ACN \rightarrow TCN$ or $ACY \rightarrow AGY$ (tv) from Jost *et al.* (2008): this site is implicated in toxin affinity by mutations engineered by Choudhary *et al.* (2003).
- 7 events of M(DIII.p4)T, $ATG \rightarrow ACG$ (ti), with 6 events from Jost *et al.* (2008) and 1 from Feldman *et al.* (2012): this replacement is implicated in decreased toxin affinity by a mutant engineered by Jost *et al.* (2008).
- 2 events of D(DIII.p5)E, $GAY \rightarrow GAR$ (tv), from Feldman *et al.* (2012): this site is implicated by replacements that affect toxin affinity in Terlau *et al.* (1991) and Choudhary *et al.* (2003), though the reported effects are modest in both studies.
- 3 events of A1529G, $GCN \rightarrow GGN$ (tv), from Jost *et al.* (2008): this replacement is implicated in modest changes in toxin affinity by a mutant engineered by Jost *et al.* (2008).
- 3 events of I1561M, $ATC \rightarrow ATG$ (tv), from Jost *et al.* (2008): this site is implicated in toxin affinity by a replacement engineered in a homolog by Geffeney *et al.* (2005).
- 3 events of D(DIV.p4)N, $GAY \rightarrow AAY$ (ti), combining both studies: this replacement is implicated in modest changes in affinity by a mutant engineered by Choudhary *et al.* (2003); other replacements at this site have greater effects.

Various targets: insecticide resistance (insects). ffrench-Constant *et al.* (2004) reviews the emergence of resistance for various classes of insecticides. Parallel replacements have been observed in regard to 3 classes that target (respectively) acetylcholinesterase, ligand-gated ion channels, and voltage-gated ion channels. The mechanism of action of these insecticides has been characterized using genetic tools in *Drosophila*, which is not itself a pest, but often is found to harbor resistant strains in affected environments.

In the first category, targeting ligand-gated ion channels, ffrench-Constant *et al.* (2000) relate that nearly all reported instances of resistance to cyclodiene pesticides (see p. 461) involve site 302 (sometimes numbered 296) of the GABA receptor known as *rdl* (resistance to dieldrin) in *Drosophila*. The link between resistance and these replacements was first established clearly by genetic association: resistance was mapped to the *rdl* locus by

classical genetics, and the specific site was implicated when 58 resistant isolates all showed a change at site 302, either $Ala \to Ser$ or $Ala \to Gly$ (ffrench-Constant *et al.*, 1993). The list of implicated species (see p. 455) is as follows:

- 9 events of A(Rdl302)S $GCG \to TCG$ (tv) in *Drosophila simulans*, *Musca domestica*, *Blatella germanica*, *Aedes aegypti*, *Myzus persicae*, *Bemisia tabaci*, *Bemisia argentifolii*, *Hypothenemus hampei*, and *Tribolium castaneum*: this replacement is implicated by genetic association, when resistance maps to a locus that has only this replacement (ffrench-Constant *et al.*, 1993).
- 2 events of A(Rdl302)G $GCG \to GGG$ (tv) in *Drosophila simulans* and *Myzus persicae*: this replacement is implicated by genetic association, due to instances in which resistance maps to a locus that has only this replacement (ffrench-Constant *et al.*, 1993).

The second category, reviewed in more detail by Soderlund (2005), involves DDT and the pyrethroid insecticides that bind to an insect sodium channel encoded by the *kdr* (knockdown resistance) gene. Table 2 of Soderlund (2005) indicates that the following replacements have occurred in parallel: M918T, T929I, L1014F, L1014S, E1533G, and D1549V. The most frequent change, L1014F, is often the sole change; the combination of L1014F and M918T is linked genetically to a highly resistant "super-kdr" phenotype (p. 13); both changes have been shown experimentally to confer pyrethroid sensitivity (p. 15; Vais *et al.*, 2000); the effect of T929I also has been verified experimentally (Vais *et al.*, 2001). Soderlund (2005) cites no experimental verification for E1533G and D1549V, which are found twice each, in two moths from different genera in the sub-family *Heliothinae*.

The L1014F change, found in 9 difference species, implicates either a $TTR \to TTY$ transversion or a $CTY \to TTY$ transition. The nucleotide mutation is often not reported even when genomic or cDNA sequences have been determined, as for the Colorado potato beetle (*Leptinotarsa decemlineata*) in Lee *et al.* (1999) or Rinkevich *et al.* (2012). The easiest approach to determining the causative mutations is to begin with the NCBI popset 312618867 for *Leptinotarsa decemlineata* from Rinkevich *et al.* (2012). The aligned dataset indicates that the L1014F change represents a CT polymorphism at site 1059 relative to the reference sequence HQ589184.1, implicating a $CTY \to TTY$ transition mutation. The polymorphism in the other instances can be determined by BLASTing this sequence against the desired species, and then examining the alignment at position 1059. This indicates that L1014F is a $TTR \to TTY$ transversion in *Anopeles gambiae*, *Blatella germanica* (as indicated by Dong (1997)), *Culex pipiens*, and *Frankliniella occidentalis* (as indicated in Fig. 2 of Forcioli *et al.* (2002)). Likewise, L1014F is a $CTY \to TTY$ transition in *Leptinotarsa decemlineata*, *Musca domestica*, *Myzus persicae*, *Plutella xylostella*, and *Haematobia irritans* (as indicated by the CTT codon at position 448 in Fig. 4 of Guerrero *et al.* (1997)).

Thus, the total set of changes implicated in Table 2 of Soderlund (2005), counting only the sites for which functional effects are confirmed, is as follows:

- 2 events of M918T $ATG \to ACG$ (ti) in *Haematobia irritans* and *Musca domestica*: the combination of this change with L1014F is linked genetically to a super-kdr phenotype; some functional effects have been verified experimentally (see Soderlund 2005).

- 2 events of T929I $ACH \rightarrow ATH$ (ti) in *Pediculus capitis* and *Plutella xylostella*: this replacement is implicated in reduced sensitivity by results reported by Vais *et al.* (2001).
- 4 events of L1014F $TTR \rightarrow TTY$ (tv) in *Anopeles gambiae*, *Blatella germanica*, *Culex pipiens*, and *Frankliniella occidentalis*: this replacement is implicated by genetic association, as it is sometimes the only change (Table 2 of Soderlund 2005); some functional effects have been verified experimentally (Vais *et al.*, 2000).
- 5 events of L1014F $CTY \rightarrow TTY$ (ti) in *Haematobia irritans*, *Leptinotarsa decemlineata*, *Musca domestica*, *Myzus persicae*, and *Plutella xylostella*: this replacement is implicated by genetic association, as it is sometimes the only change (Table 2 of Soderlund 2005); some functional effects have been verified experimentally (Vais *et al.*, 2000).
- 2 events of L1014S $TTR \rightarrow TCR$ (ti) in *Anopeles gambiae* and *Culex pipiens*: this replacement is implicated by genetic association, as it is sometimes the only change (Table 2 of Soderlund 2005).

The third category cited by ffrench-Constant *et al.* (2004) involves the resistance of insects to organophosphates and carbamates via changes in acetylcholinesterase. They cite the work of Weill *et al.* (2003), which establishes that the same G119S mutation is found in resistant strains of *Anopheles gambiae*, *Culex pipiens pipiens* and *Culex pipiens quinquefasciatus*; a haplotype analysis indicates that the latter two are independent mutations rather than a case of introgression. The link between G119S and resistance established genetically (it is sometimes the only replacement distinguishing sensitive and resistant alleles) was verified by genetic engineering in *Drosophila* by Weill *et al.* (2003).

- 3 events of G119S $GGY \rightarrow AGY$ (ti) in *Anopheles gambiae*, *Culex pipiens pipiens* and *Culex pipiens quinquefasciatus*: this replacement is linked genetically to resistance as it sometimes occurs alone in resistant alleles (ffrench-Constant *et al.*, 2004); effects on sensitivity were verified by genetic engineering in *Drosophila* (Weill *et al.*, 2003).

Note that ffrench-Constant *et al.* (2004) discusses a few other kinds of resistance that do not involve parallel replacements.

ACCase: resistance to herbicides (grasses). Certain herbicides (aryloxyphenoxypropionates and cyclohexanediones) target grasses via their inhibitory effect on plastid acetyl coenzyme A carboxylase (ACCase). Liu *et al.* (2007) summarize previous reports of resistance via ACCase replacements, and examine 9 populations of Australian wild oats (*Avena sterilis* ssp. ludoviciana Durieu) finding I2041N, W2027C, and D2078G among herbicide-resistant strains. Some of these were found in multiple populations: we count them only once due to the possibility of introgression.

Liu *et al.* (2007) used an engineered wheat ACCase gene expressed in yeast to test the effect of these 3 mutations as well as the previously reported I1781L and G2096A, finding that each one by itself can confer herbicide resistance (their Fig. 3). This tabulation is extended by two additional paths and a handful of additional events in Table 1 of Beckie *et al.* (2012).

- 7 events of I1781L $ATH \rightarrow CTHorTTA$ (tv) in *Avena sterilis ssp. ludoviciana*, *Alopecurus myosuroides*, *Lolium rigidum*, *Setaria viridis*, *Avena fatua*, *Lolium multi-*

florum, and *Triticum aestivum*: this replacement is implicated by a mutant engineered by Liu *et al.* (2007).

- 2 events of W1999C $TGG \rightarrow TGY$ (tv) in *Avena sterilis* and *Avena fatua*: this replacement is implicated by a mutant engineered by Liu *et al.* (2007).
- 4 events of W2027C $TGG \rightarrow TGY$ (tv) in *Avena sterilis ssp. ludoviciana*, *Avena fatua*, *Lolium rigidum* and *Alopecurus myosuroides*: this replacement is implicated by a mutant engineered by Liu *et al.* (2007)
- 5 events of I2041N $ATH \rightarrow AAH$ (tv) in *Avena sterilis ssp. ludoviciana*, *Avena fatua*, *Alopecurus myosuroides*, *Lolium rigidum* and *Phalaris paradoxa*: this replacement is implicated by a mutant engineered by Liu *et al.* (2007).
- 5 events of D2078G $GAY \rightarrow GGY$ (ti) in *Avena sterilis ssp. ludoviciana*, *Lolium rigidum*, *Lolium multiflorum*, *Phalaris paradoxa* and *Alopecurus myosuroides*: this replacement is implicated by a mutant engineered by Liu *et al.* (2007).
- 2 events of C2088R $TGY \rightarrow CGY$ (ti) in *Lolium rigidum* and *Avena fatua*: this replacement is genetically associated with resistance by virtue of being the only replacement consistently distinguishing sensitive and resistant alleles in Yu *et al.* (2007).

ATPα1: resistance to glycosides (animals). Some naturally occurring glycoside toxins target the sodium pump ATPα1. These include cardenolides produced by milkweed and other members of the dogbane family (*Apocynaceae*), as well as cardiac glycosides produced by amphibians. Some insects have resistance allowing them to eat *Apocynaceae*; species such as monarch butterflies (*Danaus plexippus*) not only consume the toxin, but sequester it so as to make themselves noxious to predators. Resistant insects typically have undergone changes in ATPα1: the effects of many specific mutations have been explored via genetic engineering followed by functional and structural analysis.

The entire set of data on ATPα1 parallelisms reported by Zhen *et al.* (2012) is illustrated in Fig. 2, based on Fig. 1 of Zhen *et al.* (2012). Yellow-shaded species consume and sequester plants producing cardenolides; grey-shaded species merely consume them. Fig. S1 of Zhen *et al.* (2012) summarizes the sources of information on functional effects of replacements. For instance, Croyle *et al.* (1997) carried out random mutagenesis and screening of ATPα1 mutants for ouabain resistance, with results implicating the T797A replacement as well as replacements at sites 111, 118 and 122.

From Fig. 2, the replacements P118A (black), N122Y (orange), I315V (magenta) and T797A (green) clearly happen twice each. The most parsimonious reconstruction for site 122 would call for 1 change to H in Hemiptera, 2 changes in Coleoptera, and either 2 changes to H, or one change plus a reversal in Lepidoptera. We count this conservatively as 4 changes. Site 111 illustrates unusual complexity. Q111L (blue) is a single transversion $CAR \rightarrow CTR$ that occurs several times. Q111V implicates 2 changes, most parsimoniously (as argued in Aardema *et al.* 2012) derived from a Q111L ancestor, i.e., $Q \rightarrow L \rightarrow V$ via $CAR \rightarrow CTR \rightarrow GTR$. Thus, the pattern in the minimal clade containing *Danaus plexippus* and *Lycorea halia* indicates $Q \rightarrow L$ (blue) in the ancestral lineage and then $L \rightarrow V$ (light-blue) in the *Danaus* ancestor. Severally equally parsimonious scenarios involve 5 changes at site 111 in the clade that includes *C. auratus* and *M. robiniae*; the scenario with the fewest parallels is shown (this entails 2 $L \rightarrow Q$ reversals in

M. robiniae and *P. versicolora* that we do not count). Q111T, another double-nucleotide change, happens twice with no evidence of intermediates: even if one could assume that both changes occurred via successive single-nucleotide replacements, the path is ambiguous ($Q \rightarrow P \rightarrow T$ or $Q \rightarrow K \rightarrow T$), therefore no parallel can be inferred.

Zhen *et al.* (2012) do not count all of these as verified adaptive parallels. Though T797A has been verified experimentally, the occurrence of 797A and 122Y in the aphid clade has no strong correlation with cardenolide consumption, as there is 1 consumer (*A. nerii*) and 1 non-consumer (*A. pisum*). Thus, though these are parallels, we follow the original authors in not counting them as genuine adaptive parallels consistent with the hypothesis that associates cardenolide utilization with resistance via changes in ATPα1.

Ujvari *et al.* (2015) report some additional parallels among mammals and squamates (lizards and snakes) that consume glycoside-bearing plants, insects, or toads. The set of replacements shown in their Fig. 2 (see also Fig. S1) includes the Q111L and N122H events in insects indicated above, 4 additional events of Q111L, and 5 other paths. One of the paths (Q111E) is seen only once, and another path with two events (Q119D) is a double-nucleotide change, thus not counted here. The G120R change that occurs 4 times in squamates is ambiguously either $GGR \rightarrow AGR$ or $GGN \rightarrow CGN$: inspecting columns 70 to 72 of the aligned sequences from Ujvari *et al.* (2015) (NCBI popset 928240786) indicates that the change in all 4 cases is $GGA \rightarrow AGA$ (ti). The remaining paths observed are Q111R $CAR \rightarrow CGR$ (4 ti) and N122D $AAY \rightarrow GAY$ (2 ti).

- 7 events of Q111L $CAR \rightarrow CTR$ (tv): this replacement is implicated as a strong resistance-conferring mutation in the random mutagenesis of Croyle *et al.* (1997).
- 2 events of L111V $CTR \rightarrow GTR$ (tv): this site is implicated by a strong resistance-conferring mutation in the random mutagenesis of Croyle *et al.* (1997).
- 4 events of Q111R $CAR \rightarrow CGR$ (ti): the combination of this and N122D confers 1000-fold resistance to ouabain (Price and Lingrel, 1988).
- 2 events of P118A $CCN \rightarrow GCN$ (tv): this site is implicated by a resistant mutant with only a replacement at this site in Croyle *et al.* (1997) and in Schultheis *et al.* 1993.
- 4 events of G120R $GGY \rightarrow AGY$ (ti): this replacement, in combination with Q111L and A119S, confers resistance in an engineered mutant (Ujvari *et al.*, 2013).
- 2 events of N122D $AAY \rightarrow GAY$ (ti): the combination of this and Q111R confers 1000-fold resistance to ouabain (Price and Lingrel, 1988).
- 4 events of N122H $AAY \rightarrow CAY$ (tv): this replacement confers resistance to ouabain when engineered in a *Drosophila* gene and expressed in human kidney cells (Holzinger and Wink, 1996).
- 2 events of I315V $ATH \rightarrow GTH$ (ti): this replacement is implicated by analysis of the engineered mutants of Qiu *et al.* (2005).

Opsins: spectral tuning (vertebrates). The spectral sensitivity of the visual pigments known as opsins has been the subject of much research reviewed by Yokoyama and Radlwimmer (2001). Early work suggested that many pigments have shifted from red (long-wavelength) to green (middle-wavelength) sensitivity multiple times by the three changes S180A (tv), Y277F (tv) and T285A (ti). However, some pigments have sensitivities that are not

predicted by this “3-sites rule”. Yokoyama and Radlwimmer (2001) argue for a “5-sites rule” to the effect that “S180A, H197Y, Y277F, T285A, and A308S shift the max of the LWS/MWS pigments toward green by 7, 28, 7, 15, and 16 nm, respectively, and the reverse changes toward red by the same amounts,” and suggest that this formula “fully explains the variation in the spectral sensitivity of all ancestral and currently known LWS/MWS pigments in vertebrates.” In this case, replacements are implicated by a quantitative model of effects accounting for the measured spectral sensitivity of natural and engineered combinations of mutants, in addition to experimental verification for some individual sites by Asenjo *et al.* (1994). The molecular basis of spectral tuning is described in more detail in later work by Yokoyama *et al.* (2008).

The phylogeny in Fig. 3 of Yokoyama and Radlwimmer (2001), showing changes marked on specific branches, indicates that S180A occurred 4 times, H197Y occurred once (ancestor of squirrel P532 and mouse P508), Y277F occurred 3 times, T285A occurred 3 times, and A308S occurred once (mouse P508; Fig. 3 indicates this erroneously as S to A rather than A to S).

While Yokoyama and Radlwimmer (2001) mention that the 3 pigments of marmoset (*Callithrix jacchus jacchus*) follow the 5-sites rule, they do not include it in their analysis. In earlier work, Shyue *et al.* (1995) showed that pigments consistent with trichromatic vision were not ancestral to new world monkeys, but emerged separately in the marmoset and the squirrel monkey (*Saimiri sciureus*). The S180A and T285A changes each occurred twice (in squirrel monkey and marmoset), while the Y277F change occurred once in squirrel monkey. The combined results are as follows:

- 6 events of S180A $TCN \rightarrow GCN$ (tv), 4 from Yokoyama and Radlwimmer (2001) and 2 from Shyue *et al.* (1995): this replacement is implicated by engineered changes by Asenjo *et al.* (1994).
- 2 events of H197Y $CAY \rightarrow TAY$ (ti) from Yokoyama and Radlwimmer (2001): this replacement is implicated by the 5-sites rule in Yokoyama and Radlwimmer (2001).
- 4 events of Y277F $TAY \rightarrow TTY$ (tv), 3 from Yokoyama and Radlwimmer (2001) and 1 from Shyue *et al.* (1995): this replacement is implicated by engineered changes by Asenjo *et al.* (1994).
- 5 events of T285A $ACN \rightarrow GCN$ (ti), 3 from Yokoyama and Radlwimmer (2001) and 2 from Shyue *et al.* (1995): this replacement is implicated by engineered changes by Asenjo *et al.* (1994).
- 2 events of A308S $GCN \rightarrow TCN$ (tv) from Yokoyama and Radlwimmer (2001): this replacement is implicated by the 5-sites rule in Yokoyama and Radlwimmer (2001).

Prestin: convergence in echolocation (mammals). Toothed whales and two different groups of bats have evolved sensitive systems for echolocation, relying both on an unusual ability to produce sound at specific frequencies, and an unusual sensitivity in detecting frequency modulation. Several past studies argued for parallel changes between bats and whales in hearing-related genes, on the basis of sequences alone (e.g., Li *et al.*, 2010; Liu *et al.*, 2010). Recently, Liu *et al.* (2014) went beyond these previous studies to carry out a genetic analysis of changes underlying convergence in biochemical properties of prestin, a motor protein that underlies the frequency sensitivity of mammalian hearing. Two key

parameters of prestin function were measured, and shown to differ systematically between echolocating and non-echolocating mammals.

The authors identified 5 parallel replacements: N7T, I384T, S392A, L566F, and N685S. The N7T replacement alone results in a significant increase in the non-linear capacitance parameter $1/\alpha$, as expected if this change contributes to the evolution of echolocation; the other four changes also increase this parameter, though the measured difference was not significant in the case of I384T. However, the I384T change significantly decreases the parameter $V_{1/2}$, consistent with a contribution to the evolution of echolocation.

To resolve whether the L566F change is a $CTY \rightarrow TTY$ transition or a $TTR \rightarrow TTY$ transversion, we examined this codon from various species included in the phylogeny in Fig. 2 of Liu *et al.* (2014):

- GTC (V) in *Homo sapiens* AF523354.1
- CTC (L) in *Mus musculus* AY024359.2
- GTC (V) in *Ailuropoda melanoleuca* XM_002928662.2
- CAC (H) in *Equus caballus* XM_005609048.2
- CTC (L) in *Sus scrofa* NM_001135963.1 (further outgroup to cetacea)
- TTC (F) in *Bos taurus* NM_001192878.1 (outgroup to cetacea)
- CTC (L) in *Balaenoptera acutorostrata* HQ176004.1 (sister to odontoceti)
- CTC (L) in *Megaptera novaeangliae* GU219841.1 (sister to odontoceti)
- CTC (L) in *Pteropus vampyrus* XM_011365195.1 (further outgroup to CF bats)
- CTT (L) in *Megaderma lyra* GU219835.1 (outgroup to CF bats)

This suggests that the authors' inference of L566 in the cetacean ancestor changing to F in the odontoceti ancestor does not have high confidence— because the amino acid at 566 is obviously variable and is actually F in *Bos taurus*, the nearest outgroup to cetacea in their tree—, but leaves no doubt that, under the L566F scenario forwarded by the authors, the pathway would be $CTY \rightarrow TTY$ in both instances. A prestin from hippopotamus (a closer outgroup to cetacea) would be useful to resolve this ambiguity.

The entire set of parallelisms is as follows:

- 3 events of N7T $AAY \rightarrow ACY$ (tv): this replacement is implicated by mutants engineered by Liu *et al.* (2014).
- 2 events of I384T $ATH \rightarrow ACH$ (ti): this replacement is implicated by mutants engineered by Liu *et al.* (2014).
- 2 events of S392A $TCN \rightarrow GCN$ (tv): this replacement is implicated by mutants engineered by Liu *et al.* (2014).
- 2 events of L566F $CTY \rightarrow TTY$ (ti): this replacement is implicated by mutants engineered by Liu *et al.* (2014).
- 2 events of N685S $AAY \rightarrow AGY$ (ti): this replacement is implicated by mutants engineered by Liu *et al.* (2014).

HIV Protease: resistance to ritonavir (HIV). Molla *et al.* (1996) analyzed HIV protease gene sequences from 41 patients who responded poorly to monotherapy using ritonavir, a protease inhibitor. A cell-culture assay using putatively resistant virus isolates showed

resistance to ritonavir in all isolates (their Table 2), with the level of resistance tending to increase with the number of mutations from 2-fold to 41-fold. The authors also report results of a second cell-based assay using engineered mutations that showed considerable noise and few significant effects for single mutants. The argument for implicating the specific set of 9 sites in their Table 1 was that variants appeared after treatment rather than in pre-treatment samples from the same patients, and that most patients that responded well to treatment (i.e., the patients with drug-sensitive virus) only had the wild-type sequence at these positions. The strength of this association is difficult to evaluate because the authors do not provide quantitative data.

Four sites experienced changes to more than one amino acid, but the authors do not provide separate counts; another change (L33F) cannot be resolved as a transition or transversion without genome sequences, which the authors do not provide. The changes that can be scored unambiguously are I54V, I84V, K20R, L90M. We use the counts of patients in which a mutation is found (last row of their Table 1), rather than the number of sequences, because sequences sampled from the same patient may have inherited the same mutation, whereas we mean to count only independent mutations.

- 25 events of I54V $ATH \rightarrow GTH$ (ti): this replacement is implicated by genetic association with resistance in Molla *et al.* (1996).
- 7 events of I84V $ATH \rightarrow GTH$ (ti): this replacement is implicated by genetic association with resistance in Molla *et al.* (1996).
- 9 events of K20R $AAR \rightarrow AGR$ (ti): this replacement is implicated by genetic association with resistance in Molla *et al.* (1996).
- 4 events of L90M $YTG \rightarrow ATG$ (tv): this replacement is implicated by genetic association with resistance in Molla *et al.* (1996).

Hemoglobin: altitude adaptation (birds). Projecto-Garcia *et al.* (2013) studied hemoglobin properties in South American hummingbirds (*Trochilidae*) from high and low altitude populations. The major isoform HbA in high-altitude populations tended to show higher oxygen affinity, resulting in a significant positive correlation of altitude and affinity. Site-directed mutagenesis confirmed that two recurrent changes, G(β^A13)S and G(β^A83)S (both implicating $GGY \rightarrow AGY$ transitions), increase affinity. The authors then surveyed β^A globin genes from 63 species, estimating that these two changes had occurred ≥ 4 and ≥ 13 times.

In a separate study of low- and high-altitude populations of Andean waterfowl (*Anatidae*), McCracken *et al.* (2009) found levels of parallelism far in excess of the expectations of a simulated null model, including 5 kinds of recurrent changes in β-hemoglobin alleles (2 events each of T4S, D94E and L133M, and 3 events each of G13S and A116S), and 2 kinds of recurrent changes in α-hemoglobin (2 events of T8A, and 5 events of A77T). However, mutational effects were not verified. A subsequent study by Natarajan *et al.* (2015) supported some of the proposed parallels, while undercutting other claims. Natarajan *et al.* (2015) use site-directed mutagenesis to show that the A(β^A116)S mutation increases affinity, and they argue that the D(β^A94)E mutation is likely to be causative for an increase in affinity, based partly on their measurements of oxygen affinity of low- and high-altitude hemoglobins, and partly based on a human mutant protein.

However, they also argue that 3 apparent parallels between the speckled teal (*Anas flavirostris*) and the yellow-billed pintail (*Anas georgica*) are due to introgression. This removes, from the above list of parallels reported by McCracken *et al.* (2009), the L(β^A133)M change, and it reduces from 3 to 2 the numbers for G(β^A13)S and A(β^A116)S. Natarajan *et al.* (2015) also provide reasons to doubt that some of the remaining changes are causative. They note in particular that A(α^A77)T occurs at a CpG mutation hotspot, and that the hemoglobins of high- and low-altitude torrent ducks (*Merganetta armata*), which differ **only** by A(α^A77)T, do not have significantly different oxygen affinities. The case of G(β^A13)S is more ambiguous: along with 2 other mutations, it distinguishes between 2 low-altitude ruddy duck (*Oxyura jamaicensis*) isoforms that do not differ in affinity, suggesting that it may be ineffectual; however, it also occurs in a high-altitude population of speckled teal, and was linked previously to functional changes by Projecto-Garcia *et al.* (2013).

Here we make the conservative assumption of including only the D94E and A116S β^A-hemoglobin parallels from McCracken *et al.* (2009), the ones for which there is evidence of effects beyond a phylogenetic pattern, combining these with the 2 parallels reported by Projecto-Garcia *et al.* (2013).

Natarajan *et al.* (2016) then presented an analysis of 56 pairs of high- and low-altitude populations or species, identifying exactly 4 functionally verified parallel pathways in β-hemoglobins, including G83S, A86S, D94E and A116S. Three of these pathways were identified earlier: the 6 events of G83S are redundant with Projecto-Garcia *et al.* (2013); the 2 events of A116S are redundant with McCracken *et al.* (2009); and 2 of 3 events of D94E are redundant with McCracken *et al.* (2009), while the case of *Metriopelia melanoptera* is new. The 2 cases of A86S $GCN \rightarrow TCN$ (tv), in *Cinclodes albiventris* and *Chloephaga melanoptera* are also novel.

The combined results of these studies implicate the following changes:

- 4 events of G13S $GGY \rightarrow AGY$ (ti) from Projecto-Garcia *et al.* (2013): this replacement is implicated by engineered mutants by Projecto-Garcia *et al.* (2013).
- 13 events of G83S $GGY \rightarrow AGY$ (ti) from Projecto-Garcia *et al.* (2013): this replacement is implicated by engineered mutants by Projecto-Garcia *et al.* (2013).
- 2 events of A86S $GCN \rightarrow TCN$ (tv), from Natarajan *et al.* (2016): this replacement is implicated by an engineered mutant in Natarajan *et al.* (2016).
- 3 events of D94E $GAY \rightarrow GAR$ (tv) from Natarajan *et al.* (2016): this replacement is implicated by genetic association (it is the only replacement distinguishing the β-hemoglobins of low- and high-affinity alleles in the crested duck) in Natarajan *et al.* (2015).
- 2 events of A116S $GCN \rightarrow TCN$ (tv) from McCracken *et al.* (2009): this replacement is implicated by a mutant engineered by Natarajan *et al.* (2015).

Ribonucleases: convergence in foregut fermentation (monkeys). Foregut fermentation of plant matter is a complex adaptation that has evolved multiple times, in colobine monkeys such as langurs, ruminants such as cows and goats, and the hoatzin, a bird. Lacking the native ability to digest cellulose, these organisms have evolved an extra chamber, the foregut, in which plant matter is digested by microbes, which are then digested down-

stream in the unusually acidic environment that results from fermentation. In a classic paper, Stewart *et al.* (1987) argued for sequence convergence in lysozymes between cows and langurs. Foregut fermentation is also associated with elevated use of acid-tolerant RNase (ribonuclease) in the small intestine, to harvest the nitrogen contained in bacteria. African and Asian leaf-eating colobine monkeys (*Colobinae*) separately evolved one or two (respectively) duplicate RNase loci that then underwent rapid evolution, including changes that lower the pH optimum of the enzyme, while retaining an RNase1 locus without an unusual pattern of evolution.

Zhang (2006) used site-directed mutagenesis and enzyme assays with constructs derived from the guereza (*Colobus guereza*), a colobine monkey, to show that the 3 changes that occurred in parallel in African- and Asian-derived RNase2 or RNase3 loci (R4Q, K6E and R39W) are sufficient to cause the observed change in pH optimum. In a later study with a greatly expanded set of species, Yu *et al.* (2010) identified 2 additional events of R39W in *Piliocolobus badius* (African) and in a clade of *Rhinopithecus* species (Asian), and 2 additional events of K6E in *P. badius* and a clade of *Trachypithecus* species (see their Fig. 6).

The R39W change is ambiguous due to Arginine having AGR and CGN codons. However, relevant RNase1 mRNA sequences (e.g., DQ516063, AF449642, NM_001284863) indicate without ambiguity that R39W is a $CGG \rightarrow TGG$ transition. Thus, the total set is as follows:

- 2 events of R4Q $CGR \rightarrow CAR$ (ti): this replacement is implicated (in combination with the other two) by a mutant engineered by Zhang (2006).
- 4 events of K6E $AAR \rightarrow GAR$ (ti): this replacement is implicated (in combination with the other two) by a mutant engineered by Zhang (2006).
- 4 events of R39W $CGG \rightarrow TGG$ (ti): this replacement is implicated (in combination with the other two) by a mutant engineered by Zhang (2006).

β-tubulin: resistance to benzimidazole (fungi and some nematodes). Benzimidazole, which acts on β-tubulin, is used in agricultural settings to control both fungal infections of crops, and worm infections in livestock. Koenraadt *et al.* (1992) isolated resistant strains of ascomycete fungi, sequenced their β-tubulin genes, and identifyied a variety of changes. Elard *et al.* (1996) later did the same with resistant worms of *Teladorsagia circumcincta.* Note that, in their Table 1, the fungal mutations as well as a *C. elegans* mutation actually trace to laboratory mutagenesis and selection rather than field isolates (e.g., see their references 15, 16 and 17). Together Koenraadt *et al.* (1992) and Elard *et al.* (1996) cite various references that isolate functional effects of individual changes, including F167Y, E198D, E198Q, E198G, E198K, and F200Y.

The tally of repeated changes with evidence of functional effects includes the following:

- 7 events of E198K $GAG \rightarrow AAG$ (ti) in *Venturia inaequalis*, *Monilinia fructicola*, *Penicillium puberulum*, *Penicillium digitatum*, *Penicillium aurantiogriseum*, *Penicillium viridicatum*, and *Sclerotinia homoeocarpa*: this replacement is implicated by the mutant selection of Jung *et al.* (1992), and by genetic association, being found repeatedly (in different species) among one or a few replacements that distinguish resistant and sensitive alleles (Koenraadt *et al.*, 1992).

- 5 events of E198A $GAG \rightarrow GCG$ (tv) in *Venturia inaequalis*, *Venturia pirina*, *Penicillium puberulum*, *Penicillium expansum*, and *Penicillium aurantiogriseum*: this replacement is implicated by genetic association, being found repeatedly (in different species) among one or a few replacements that distinguish resistant and sensitive alleles (Koenraadt *et al.*, 1992).
- 6 events of F200Y $TTY \rightarrow TAY$ (tv) in the nematodes *Haemonchus contortus* and *Teladorsagia circumcincta*, and in the fungi *Venturia inaequalis*, *Venturia pirina*, *Penicillium italicum* and *Penicillium aurantiogriseum*: this replacement is implicated in resistance by the mutation engineered by Kwa *et al.* (1995).

References for Supplementary Text

Aardema, M. L. and Andolfatto, P. 2016. Phylogenetic incongruence and the evolutionary origins of cardenolide-resistant forms of Na+ ,K+-ATPase in *Danaus* butterflies. *Evolution*, 70(8): 1913–21.

Asenjo, A. B., Rim, J., and Oprian, D. D. 1994. Molecular determinants of human red/green color discrimination. *Neuron*, 12(5): 1131–8.

Beckie, H. J., Warwick, S. I., and Sauder, C. A. 2012. Basis for Herbicide Resistance in Canadian Populations of Wild Oat (Avena fatua). *Weed Science*, 60(1): 10–18.

Bricelj, V. M., Connell, L., Konoki, K., Macquarrie, S. P., Scheuer, T., Catterall, W. A., and Trainer, V. L. 2005. Sodium channel mutation leading to saxitoxin resistance in clams increases risk of PSP. *Nature*, 434(7034): 763–7.

Bull, J. J., Badgett, M. R., Wichman, H. A., Huelsenbeck, J. P., Hillis, D. M., Gulati, A., Ho, C., and Molineux, I. J. 1997. Exceptional convergent evolution in a virus. *Genetics*, 147(4): 1497–507.

Choudhary, G., Yotsu-Yamashita, M., Shang, L., Yasumoto, T., and Dudley, S. C., J. 2003. Interactions of the C-11 hydroxyl of tetrodotoxin with the sodium channel outer vestibule. *Biophys J*, 84(1): 287–94.

Crill, W. D., Wichman, H. A., and Bull, J. J. 2000. Evolutionary reversals during viral adaptation to alternating hosts. *Genetics*, 154(1): 27–37.

Croyle, M. L., Woo, A. L., and Lingrel, J. B. 1997. Extensive random mutagenesis analysis of the Na+/K+-ATPase alpha subunit identifies known and previously unidentified amino acid residues that alter ouabain sensitivity–implications for ouabain binding. *Eur J Biochem*, 248(2): 488–95.

Dong, K. 1997. A single amino acid change in the para sodium channel protein is associated with knockdown-resistance (kdr) to pyrethroid insecticides in German cockroach. *Insect Biochem Mol Biol*, 27(2): 93–100.

Elard, L., Comes, A. M., and Humbert, J. F. 1996. Sequences of beta-tubulin cDNA from benzimidazole-susceptible and -resistant strains of *Teladorsagia circumcincta*, a nematode parasite of small ruminants. *Mol Biochem Parasitol*, 79(2): 249–53.

Feldman, C. R., Brodie, E. D., Jr., Brodie, E. D.,III, and Pfrender, M. E. 2012. Constraint shapes convergence in tetrodotoxin-resistant sodium channels of snakes. *Proc Natl Acad Sci U S A*, 109(12): 4556–61.

ffrench-Constant, R. H., Steichen, J. C., Rocheleau, T. A., Aronstein, K., and Roush, R. T. 1993. A single-amino acid substitution in a gamma-aminobutyric acid subtype A receptor locus is associated with cyclodiene insecticide resistance in *Drosophila* populations. *Proc Natl Acad Sci U S A*, 90(5): 1957–61.

ffrench-Constant, R. H., Anthony, N., Aronstein, K., Rocheleau, T., and Stilwell, G. 2000. Cyclodiene insecticide resistance: from molecular to population genetics. *Annu Rev Entomol*, 45: 449–66.

ffrench-Constant, R. H., Daborn, P. J., and Le Goff, G. 2004. The genetics and genomics of insecticide resistance. *Trends Genet*, 20(3): 163–70.

Forcioli, D., Frey, B., and Frey, J. E. 2002. High nucleotide diversity in the para-like voltage-sensitive sodium channel gene sequence in the western flower thrips (Thysanoptera: Thripidae). *J Econ Entomol*, 95(4): 838–48.

Geffeney, S. L., Fujimoto, E., Brodie, E. D., r., Brodie, E. D., J., and Ruben, P. C. 2005. Evolutionary diversification of TTX-resistant sodium channels in a predator-prey interaction. *Nature*, 434(7034): 759–63.

Guerrero, F. D., Jamroz, R. C., Kammlah, D., and Kunz, S. E. 1997. Toxicological and molecular characterization of pyrethroid-resistant horn flies, *Haematobia irritans*: identification of kdr and super-kdr point mutations. *Insect Biochem Mol Biol*, 27(8-9): 745–55.

Holzinger, F. and Wink, M. 1996. Mediation of cardiac glycoside insensitivity in the monarch butterfly (Danaus plexippus): Role of an amino acid substitution in the ouabain binding site of Na+,K+-ATPase. *J Chem Ecol*, 22(10): 1921–1937.

Jost, M. C., Hillis, D. M., Lu, Y., Kyle, J. W., Fozzard, H. A., and Zakon, H. H. 2008. Toxin-resistant sodium channels: parallel adaptive evolution across a complete gene family. *Mol Biol Evol*, 25(6): 1016–24.

Jung, M. K., Wilder, I. B., and Oakley, B. R. 1992. Amino acid alterations in the benA (beta-tubulin) gene of *Aspergillus nidulans* that confer benomyl resistance. *Cell Motil Cytoskeleton*, 22(3): 170–4.

Koenraadt, H., Somerville, S. C., and Jones, A. 1992. Characterization of mutations in the beta-tubulin gene of benomyl-resistant field strains of *Venturia inaequalis* and other plant pathogenic fungi. *Phytopathology*, 82: 1348–1354.

Kwa, M. S., Veenstra, J. G., Van Dijk, M., and Roos, M. H. 1995. Beta-tubulin genes from the parasitic nematode *Haemonchus contortus* modulate drug resistance in *Caenorhabditis elegans*. *J Mol Biol*, 246(4): 500–10.

Lee, S. H., Dunn, J. B., Marshall Clark, J., and Soderlund, D. M. 1999. Molecular analysis of kdr-like resistance in a permethrin-resistant strain of Colorado potato beetle. *Pesticide Biochemistry and Physiology*, 63(2): 63–75.

Li, Y., Liu, Z., Shi, P., and Zhang, J. 2010. The hearing gene Prestin unites echolocating bats and whales. *Current Biology*, 20(2): R55–6.

Liao, H., McKenzie, T., and Hageman, R. 1986. Isolation of a thermostable enzyme variant by cloning and selection in a thermophile. *Proc Natl Acad Sci U S A*, 83(3): 576–80.

Liu, W., Harrison, D. K., Chalupska, D., Gornicki, P., O'Donnell C, C., Adkins, S. W., Haselkorn, R., and Williams, R. R. 2007. Single-site mutations in the carboxyltransferase domain of plastid acetyl-CoA carboxylase confer resistance to grass-specific herbicides. *Proc Natl Acad Sci U S A*, 104(9): 3627–32.

Liu, Y., Cotton, J. A., Shen, B., Han, X., Rossiter, S. J., and Zhang, S. 2010. Convergent sequence evolution between echolocating bats and dolphins. *Curr Biol*, 20(2): R53–4.

Liu, Z., Qi, F. Y., Zhou, X., Ren, H. Q., and Shi, P. 2014. Parallel sites implicate functional convergence of the hearing gene prestin among echolocating mammals. *Mol Biol Evol*, 31(9): 2415–24.

MacLean, R. C., Perron, G. G., and Gardner, A. 2010. Diminishing returns from beneficial mutations and pervasive epistasis shape the fitness landscape for rifampicin resistance in *Pseudomonas aeruginosa*. *Genetics*, 186(4): 1345–54.

McCracken, K. G., Barger, C. P., Bulgarella, M., Johnson, K. P., Sonsthagen, S. A., Trucco, J., Valqui, T. H., Wilson, R. E., Winker, K., and Sorenson, M. D. 2009. Parallel evolution in the major haemoglobin genes of eight species of Andean waterfowl. *Mol Ecol*, 18(19): 3992–4005.

Meyer, J. R., Dobias, D. T., Weitz, J. S., Barrick, J. E., Quick, R. T., and Lenski, R. E. 2012. Repeatability and contingency in the evolution of a key innovation in phage lambda. *Science*, 335(6067): 428–32.

Molla, A., Korneyeva, M., Gao, Q., Vasavanonda, S., Schipper, P. J., Mo, H. M., Markowitz, M., Chernyavskiy, T., Niu, P., Lyons, N., Hsu, A., Granneman, G. R., Ho, D. D., Boucher, C. A. B., Leonard, J. M., Norbeck, D. W., and Kempf, D. J. 1996. Ordered accumulation of mutations in HIV protease confers resistance to ritonavir. *Nature Medicine*, 2(7): 760–766.

Natarajan, C., Projecto-Garcia, J., Moriyama, H., Weber, R. E., Munoz-Fuentes, V., Green, A. J., Kopuchian, C., Tubaro, P. L., Alza, L., Bulgarella, M., Smith, M. M., Wilson, R. E., Fago, A., McCracken, K. G., and Storz, J. F. 2015. Convergent evolution of hemoglobin function in high-altitude Andean waterfowl involves limited parallelism at the molecular sequence level. *PLoS Genet*, 11(12): e1005681.

Natarajan, C., Hoffmann, F. G., Weber, R. E., Fago, A., Witt, C. C., and Storz, J. F. 2016. Predictable convergence in hemoglobin function has unpredictable molecular underpinnings. *Science*, 354(6310): 336–339.

Price, E. M. and Lingrel, J. B. 1988. Structure-function relationships in the Na,K-ATPase alpha subunit: site-directed mutagenesis of glutamine-111 to arginine and asparagine-122 to aspartic acid generates a ouabain-resistant enzyme. *Biochemistry*, 27(22): 8400–8.

Projecto-Garcia, J., Natarajan, C., Moriyama, H., Weber, R. E., Fago, A., Cheviron, Z. A., Dudley, R., McGuire, J. A., Witt, C. C., and Storz, J. F. 2013. Repeated elevational transitions in hemoglobin function during the evolution of Andean hummingbirds. *Proc Natl Acad Sci U S A*, 110(51): 20669–74.

Qiu, L. Y., Krieger, E., Schaftenaar, G., Swarts, H. G., Willems, P. H., De Pont, J. J., and Koenderink, J. B. 2005. Reconstruction of the complete ouabain-binding pocket of Na,K-ATPase in gastric H,K-ATPase by substitution of only seven amino acids. *J Biol Chem*, 280(37): 32349–55.

Rinkevich, F. D., Su, C., Lazo, T. A., Hawthorne, D. J., Tingey, W. M., Naimov, S., and Scott, J. G. 2012. Multiple evolutionary origins of knockdown resistance (kdr) in pyrethroid-resistant Colorado potato beetle, *Leptinotarsa decemlineata*. *Pesticide Biochemistry and Physiology*, 104(3): 192–200.

Rokyta, D. R., Joyce, P., Caudle, S. B., and Wichman, H. A. 2005. An empirical test of the mutational landscape model of adaptation using a single-stranded DNA virus. *Nat Genet*, 37(4): 441–4.

Schultheis, P. J., Wallick, E. T., and Lingrel, J. B. 1993. Kinetic analysis of ouabain binding to native and mutated forms of Na,K-ATPase and identification of a new region involved in cardiac glycoside interactions. *J Biol Chem*, 268(30): 22686–94.

Shyue, S. K., Hewett-Emmett, D., Sperling, H. G., Hunt, D. M., Bowmaker, J. K., Mollon, J. D., and Li, W. H. 1995. Adaptive evolution of color vision genes in higher primates. *Science*, 269(5228): 1265–7.

Soderlund, D. 2005. *Sodium Channels*, volume 5 of *Comprehensive Molecular Insect Science*. Elsevier, New York.

Stewart, C. B., Schilling, J. W., and Wilson, A. C. 1987. Adaptive evolution in the stomach lysozymes of foregut fermenters. *Nature*, 330(6146): 401–4.

Terlau, H., Heinemann, S. H., Stuhmer, W., Pusch, M., Conti, F., Imoto, K., and Numa, S. 1991. Mapping the site of block by tetrodotoxin and saxitoxin of sodium channel II. *FEBS Lett*, 293(1-2): 93–6.

Ujvari, B., Mun, H. C., Conigrave, A. D., Bray, A., Osterkamp, J., Halling, P., and Madsen, T. 2013. Isolation breeds naivety: island living robs Australian varanid lizards of toad-toxin immunity via four-base-pair mutation. *Evolution*, 67(1): 289–94.

Ujvari, B., Casewell, N. R., Sunagar, K., Arbuckle, K., Wuster, W., Lo, N., O'Meally, D., Beckmann, C., King, G. F., Deplazes, E., and Madsen, T. 2015. Widespread convergence in toxin resistance by predictable molecular evolution. *Proc Natl Acad Sci U S A*, 112(38): 11911–6.

Vais, H., Williamson, M. S., Goodson, S. J., Devonshire, A. L., Warmke, J. W., Usherwood, P. N., and Cohen, C. J. 2000. Activation of Drosophila sodium channels promotes modification by deltamethrin. Reductions in affinity caused by knock-down resistance mutations. *J Gen Physiol*, 115(3): 305–18.

Vais, H., Williamson, M. S., Devonshire, A. L., and Usherwood, P. N. 2001. The molecular interactions of pyrethroid insecticides with insect and mammalian sodium channels. *Pest Manag Sci*, 57(10): 877–88.

Venkatesh, B., Lu, S. Q., Dandona, N., See, S. L., Brenner, S., and Soong, T. W. 2005. Genetic basis of tetrodotoxin resistance in pufferfishes. *Curr Biol*, 15(22): 2069–72.

Weill, M., Lutfalla, G., Mogensen, K., Chandre, F., Berthomieu, A., Berticat, C., Pasteur, N., Philips, A., Fort, P., and Raymond, M. 2003. Comparative genomics: insecticide resistance in mosquito vectors. *Nature*, 423(6936): 136–7.

Yokoyama, S. and Radlwimmer, F. B. 2001. The molecular genetics and evolution of red and green color vision in vertebrates. *Genetics*, 158(4): 1697–710.

Yokoyama, S., Yang, H., and Starmer, W. T. 2008. Molecular basis of spectral tuning in the red- and green-sensitive (M/LWS) pigments in vertebrates. *Genetics*, 179(4): 2037–43.

Yu, L., Wang, X. Y., Jin, W., Luan, P. T., Ting, N., and Zhang, Y. P. 2010. Adaptive evolution of digestive RNASE1 genes in leaf-eating monkeys revisited: new insights from ten additional colobines. *Mol Biol Evol*, 27(1): 121–31.

Yu, Q., Collavo, A., Zheng, M. Q., Owen, M., Sattin, M., and Powles, S. B. 2007. Diversity of acetyl-coenzyme A carboxylase mutations in resistant Lolium populations: evaluation using clethodim. *Plant Physiol*, 145(2): 547–58.

Zhang, J. 2006. Parallel adaptive origins of digestive RNases in Asian and African leaf monkeys. *Nat Genet*, 38(7): 819–23.

Zhen, Y., Aardema, M. L., Medina, E. M., Schumer, M., and Andolfatto, P. 2012. Parallel molecular evolution in an herbivore community. *Science*, 337(6102): 1634–7.
